# Politics, Pandemics, and Trauma: Understanding and Addressing Latino Health Needs Through a Culturally-Informed Lens

**DOI:** 10.3389/fpubh.2022.877328

**Published:** 2022-07-15

**Authors:** Mary Lehman Held, Tatiana Villarreal-Otálora, Jane McPherson, Porter Jennings-McGarity

**Affiliations:** ^1^College of Social Work, University of Tennessee, Knoxville, Knoxville, TN, United States; ^2^Department of Social Work and Human Services, Kennesaw State University, Kennesaw, GA, United States; ^3^School of Social Work, University of Georgia, Athens, GA, United States; ^4^PACEs Connection, TSNE Missionworks, Boston, MA, United States

**Keywords:** Latino immigrant communities, trauma, Southeastern U.S, immigration policies, COVID-19, service provision

## Abstract

Latino communities in the United States (U.S.) have long endured trauma due to multiple intersecting social and political forces. New restrictive immigration policies since 2016 and the COVID-19 pandemic have each created novel stressors for Latino communities, while escalating the risk of mental health disorders and highlighting the communities' vulnerabilities. The effects of these stressors have been particularly pronounced in southeastern states, such as Tennessee and Georgia, due to their state-level anti-immigrant legislation. Yet, we lack sufficient data to understand how these factors present among Latinos seeking services. To focus attention on the specific experiences of Latino communities living in the U.S. Southeast, the authors analyzed the perspectives of 44 service providers working with these communities in the region using qualitative data collected in an online survey administered during the COVID-19 pandemic and while President Trump's exclusionary immigration policies were in force. Four themes were identified: (1) Latino communities' strengths; (2) impact of the Trump administration on Latino communities; (3) impact of COVID-19's on Latino communities; and (4) strategies to enhance service delivery in Latino communities. Results provide meaningful data to inform micro- and macro-level service delivery in two exclusionary policy states and beyond. Findings suggest future research should include other new immigrant destinations and explore perceptions of Latino community members.

## Introduction

In the U.S., exclusionary immigration policies and anti-immigrant political propaganda are stressors that have long created anxiety and trauma among Latino^1^ communities. Since 2016, increasingly restrictive policies and inflammatory anti-immigrant rhetoric have required Latinos to cope with intensified levels of immigration-related trauma, to which COVID-19 has more recently presented novel and painful stressors (1–3). Further, much of the anti-immigrant sentiment under former President Trump specifically targeted Mexican and Central American communities, exacerbating anxieties in Latino communities (4–6). As such, the increased focus on exclusionary policies and Immigration and Customs Enforcement (ICE) efforts cultivated a climate of fear and oppression (7–9). The pandemic further exacerbated stress within Latino communities due to disproportionate rates of both COVID diagnosis and detrimental financial impacts associated with pandemic-related lost wages (10–12). These factors collectively create a challenging new landscape, with Latino individuals simultaneously experiencing increased mental health needs and heightened barriers to care (3, 13–17). In Tennessee and Georgia where anti-immigrant policies at the state and local levels create perilous environments for Latino communities, these exclusionary policies and the pandemic each create intense stress for affected families (18).

Health care providers can serve a vital role in reaching Latino communities and detecting and treating mental health needs. Yet, providers in the Southeast may not be fully prepared to engage and serve Latino clients (19, 20). In response, the goal of this article is twofold: (1) to delineate key service needs and service access barriers among Latinos, with a particular focus on Tennessee and Georgia, and (2) to detail empirical strategies that providers can use to reduce these barriers and to strengthen culturally-informed service provision for Latino clients. Toward these goals, qualitative responses from a study of providers serving Latino communities in Tennessee and Georgia are presented and further expanded on in relation to existing literature.

## Latino Communities in the Southeast

Since 2012, Latinos have constituted more than half of the population growth in the U.S. overall, with the highest growth–a 26% increase between 2010 and 2019–in the U.S. Southeast (21, 22). This growth reflects a shifting settlement pattern, as Latinos increasingly move to new destination settlement states, such as those in the Southeast (23–25). “New destination” was coined to describe those states that, prior to the 1990's, were home to few Latinos and have since experienced rapid Latino population growth.

The Southeastern U.S. contains many of these new Latino destinations. Latinos' social mobility and well-being are often stunted in new destinations, as compared to established ones, for several reasons (26). First, southeastern states largely lack established Latino communities that possess the historical and generational knowledge of traditional destinations, like California, Texas, and New York (27). As such, newly-arrived Latinos in the Southeast have fewer informal supports to guide their navigation of and integration within their new homes. Second, health and social service providers in southeastern states often have less training and experience to aid in understanding mental health service needs and appropriate interventions through a linguistically competent or culturally-informed lens (19, 20, 28). Finally, the southeastern region of the U.S. is characterized by punitive local immigration enforcement policies, hostile anti-immigrant perspectives among the general public, and a lived experience of political and social discrimination reported by Latinos (26, 27, 29–31).

### Tennessee and Georgia

Two southeastern states, Tennessee and Georgia, are key examples of locations with growing Latino communities that have exclusionary immigration policies and insufficient support for Latinos contending with trauma and mental health needs. Exclusionary immigration policies refer to federal- and state-level legislation that excludes immigrants from access to services (e.g., public benefits) or from participating in aspects of life (e.g., driving) in the U.S. (18, 32, 33).

#### Tennessee

Tennessee is home to nearly 400,000 Latinos, representing about 6% of the state's population and 43% of Tennessee's immigrants (34, 35). In the past 10 years, 65.2% of population growth in Tennessee is accounted for by Latinos, a significant portion of whom are undocumented or underdocumented. Undocumented immigrants do not have legal permission to be in the U.S. In contrast, underdocumented immigrants often have a form of legal status (e.g., Deferred Action of Childhood Arrivals [DACA]) or Temporary Protected Status [TPS]) that can expire with little notice and fails to provide a pathway to legal citizenship. For example, when President Trump ended DACA and suspended TPS for some countries, immigrants with those protections were suddenly undocumented and at risk of deportation. Many Latino families include U.S. citizens, as well as family members who are under- or undocumented. These “mixed status” families are especially vulnerable to state- and county-level anti-immigrant policies that inflict daily stress and that can detrimentally impact physical and mental health. For example, of the 95 counties in Tennessee, only one upholds “sanctuary city” practices, meaning that only one county limits local cooperation with ICE by, for example, prohibiting ICE detainers (i.e., holding individuals on behalf of ICE for up to 48 hours) without a court order or warrant (36, 37). Yet, even this county cannot take an official sanctuary policy stance, as Tennessee House Bill 2315 prohibits any sanctuary policies in the state (38). Of the remaining counties, two passed the anti-immigrant 287(g) policy deputizing local law enforcement officers to arrest and detain undocumented immigrants on behalf of ICE (39).

Tennessee upholds multiple additional policies that create stress for Latino communities. Not only are undocumented immigrants unable to obtain driver's licenses in the state, but Tennessee seriously considered legislation that would have prohibited driver's license and permit examinations from being translated to non-English languages (40). Though not passed, proposed legislation both sets and reflects the state's sociopolitical context. Existing exclusionary policies also affect employment, with House Bill 1378 mandating employers with at least 50 employees to use E-Verify, a federal government tool to confirm that employees are legally permitted to work (41). In regard to education, undocumented immigrants must pay out-of-state tuition at public universities. A bill was proposed in 2015 to allow in-state tuition but failed to pass by one vote (42).

Despite the additional need for health and social services related to the exclusionary policies, Latinos also encounter substantial barriers to accessing care in Tennessee. For example, one state policy requires all immigrants to wait 5 years before accessing Medicaid or the Children's Health Insurance Program [CHIP; (43)]. This policy is especially penalizing for pregnant women and children. Even when Latinos overcome the barriers and seek care, providers are often not sufficiently trained to deliver culturally-informed or linguistically-appropriate services (19, 20). For example, in 2020, only 33% of Tennessee's mental health facilities were reported to have a Spanish-speaking provider (17). A 2022 review of *Psychology Today'*s database showed that of the 3,028 providers registered in Tennessee, only 59 (just under 2%) advertised Spanish proficiency. Given this lack of appropriate providers, perhaps it is unsurprising that recent data from SAMHSA (44) found that only 1% of individuals utilizing mental health services in Tennessee were Latino. The high number of access barriers may well be reflected in low service utilization rates.

#### Georgia

On the cusp of becoming a majority-minority state, Georgia has a diversity index of 64%, ranking 9th in the U.S. for racial and ethnic diversity (45). Much of its diversity is fueled by the Latino population, which grew by 32% in the last decade and is projected to increase by 45% by 2065 (45). Recent statistics show that Latinos represent 11% of Georgia's total population and 38% of the state's immigrant population (45, 46).

For decades, Georgia has heavily relied on the influx of Latino immigrants, predominantly of Central American descent, for labor. Prior to the 1980's, the influx was temporary and characterized by single men who would return to their native countries after working seasonally (47). However, in the late 1980's, with a rise in rhetoric suggesting that individuals from Mexico were taking employment opportunities from White European-Americans in the U.S., the 1986 Immigration Reform and Control Act (48) was passed, paradoxically spiking the Latino population within the state. IRCA severely limited a worker's ability to cross the U.S. border for temporary work, thereby forcing many Latino immigrants to settle permanently in the U.S., rather than return home without employment. Immigrants who came as temporary workers and stayed became “undocumented,” though many continued to work. In the 1990's, the Latino population in Georgia spiked again as Atlanta-city planners informally recruited and welcomed their labor in preparation for the 1996 Olympics (47).

While Latino families have been informally welcomed by Georgia business owners, they have simultaneously experienced discrimination from their non-Latino neighbors. Discrimination against Latinos in Georgia is propagated through multiple domains, including education, employment, transportation, civic engagement, state assistance, and health (18). Policies directly aimed at limiting Latino immigrants' livelihood initially focused on restricting their mobility through amending the Georgia driver's license requirements in 2005 to exclude undocumented individuals. A year later, the Georgia Security and Immigration Compliance Act (49) mandated that all public employers verify an individual's legal status using E-Verify prior to hiring; this mandate was expanded to private employers in 2011. Such restrictive state-level policies severely hindered Latino immigrants' economic stability and mobility in Georgia.

Furthermore, such restrictive state-level policies adversely impact the educational aspirations and overall mental health of Latino immigrants in Georgia (18). For example, the Georgia Board of Regents Policy 4.1.6 and 4.3.4, together effectively block undocumented immigrants from attending post-secondary institutions in Georgia [see McPherson et al. (50), for further information]. In addition to living with the uncertainty of how to access post-secondary education, many Latino immigrant children also live under a constant state of fear due to the state's detention and deportation policies. For instance, two additional Georgia counties and the Georgia Department of Corrections recently signed on to the 287(g) program resulting in a total of five Georgia counties entering into this written agreement with the Department of Homeland Security (39). The state's anti-immigrant sentiments are reflected in the fact that Georgia houses two ICE processing centers and two ICE detention centers. One of Georgia's ICE detention centers is currently the largest in the U.S., averaging more than 1,000 detainees per day, and is set to expand (51).

Anti-immigrant policies and sentiments, primarily directed toward Latinos, are so rampant in Georgia that the state is considered one of the most exclusionary immigrant climates within the U.S. (18). For example, anti-Latino hate crimes have increased by more than 100% in Georgia in the last decade (52). The traumatic and stressful effects of this hostile immigrant climate are reflected in Latinos' standard of living and health. It is estimated that nearly 30% of Latino children in Georgia live below the poverty line (53) and that nearly 20% are struggling with a mental health issue (54). Unfortunately, 16% of Latino children in Georgia are uninsured (55) and cannot seek treatment due to cost or fear of deportation. Thus, many Latino families in Georgia are left to rely heavily on emergency room settings for care. For example, in the first 6 months after discharge, 13% of Latino psychiatric patients sought emergency room follow-up rather than utilizing outpatient mental health services (56). In addition to cost and fear-related treatment barriers, there is a scarcity of Latino-focused agencies and bilingual service providers in Georgia with only 20% of their mental health facilities providing services in Spanish (17). Overall, per a review of Psychology Today's 2022 database, only 3.3% of the 5,730 providers registered in Georgia provide mental healthcare services in Spanish. The scant number of bilingual mental health providers explains the lack of accredited bilingual outpatient mental healthcare agencies in Georgia. As of writing this manuscript, the Georgia Department of Community Health-Healthcare Facility Regulation Board has accredited only two bilingual—Spanish and English—multicultural agencies in the state to provide outpatient mental healthcare services (57).

## Methods

### Participants and Procedures

To gain insight into provider-perceived experiences of Latino communities in the Southeast, an online cross-sectional survey study was administered in early 2020 using targeted and snowball sampling strategies. Using site-based (58) and respondent-driven advertisement (59), adult providers delivering health, mental health, psychosocial, legal, and educational services to Latinos in Georgia and Tennessee were recruited to participate [For more details regarding the survey's ethics board approval and methodology, please see Held et al. (19)]. Four open-ended questions exploring Latino communities' strengths, evolving needs since the 2016 election and the COVID-19 pandemic, and service utilization barriers were included in the survey. Of 109 study participants, 44 responded to the open-ended questions. Most respondents were female (*n* = 35), nearly half (*n* = 20) identified as Latino, and more worked in Georgia (*n* = 28) than in Tennessee (*n* = 16). On average, the respondents were in their late thirties (*M* = 38; range 23 - 62). The sample was interdisciplinary, with respondents representing policy (n = 1), public health (*n* = 2), legal services (*n* = 5), counseling (*n* = 5), and social work (*n* = 13), among other fields.

### Data Analysis

The data were imported into NVivo (60) and examined using applied thematic analysis (61) to explore participants' responses and extract patterns. The analysis occurred in three stages. First, a structural codebook was developed, guided by the structure of the open-ended questions. This structural guide included participants' perceptions of (1) changes for their Latino clients due to the complex U.S. political climate since 2016; (2) changes due to the pandemic; (3) Latino client strengths; and (4) individual and organizational strategies to build trust with Latino clients. The second stage consisted of adding inductive codes to the structurally coded texts and thus evaluating and revising the codebook. This iterative process occurred through a comprehensive reading of half of the participant responses. Once code saturation was reached [i.e., no additional revisions nor additions made to the codebook; (61)], which occurred after the analysis of ten participant responses, the final stage began. This final stage consisted of assigning the finalized inductive codes to the structurally coded data and reviewing them for patterns, commonalities, and exceptions.

## Results

The data analysis provided four overarching themes: (1) Latino communities' strengths; (2) impactof the Trump administration; (3) Impact of COVID-19; and (4) Strategies to enhance service delivery to Latino communities. These themes were organized to improve understanding of provider-perceived strengths within Latino communities, the impacts of two large-scale stressors on Latino communities, and approaches for designing and delivering effective mental health services.

### Latino Communities' Strengths

This first theme highlights strengths observed among Latino clients. These strengths included resilience, familism, community, and ethnic pride. The majority (60%; *n* = 27) of participants used the word “resilience” when referring to Latino community strengths. One participant's response summarized resilience specific to the recent sociopolitical climate:

I have a lot of respect for how challenging my Latino clients' lives are with issues not limited to mental health but general issues of safety, political and social discrimination, and living in fear. Yet, despite increasingly harsh circumstances, they have this endurance and determination to make a good living for themselves and their families and overcome adversities. It is admirable.

Another referenced strength was familism, which reflects a high value of relationships with immediate and extended family members, as well as close friends (62). One participant indicated that familism is evident in service-seeking behaviors among Latino clients, “they [the identified clients] usually present with other family members (siblings, extended relatives) to provide them with support.” Participants discussed family as a broad construct composed of Latinos' community members regardless of blood or marital connection, which is uniquely reflective of the cultural strength of familism (62). The strength of community was also noted, with one participant stating, “they [Latino clients] eagerly help out those around them and see everyone as part of their larger community, each sharing what they know with one another.” Lastly, ethnic pride also was identified by the providers as a valued strength. For example, one participant emphasized the value of Latino clients having a sense of ethnic pride in their Latino clients “wanting to share their culture.”

### Impact of the Trump Administration

For the second theme, participants emphasized the presence of fear and anxiety as key factors associated with the Trump administration. Participants' comments underscored an escalation of fear among Latino clients during the Trump administration. A participant wrote:

The Latino/a immigrant experience has definitely changed due to a new surge of fear regarding both erratic federal immigration policies and general hate from anti-immigrant community members whose ideas, words, and actions have been reinforced by the Trump administration's anti-immigrant rhetoric.

Further, participants pointed out that this fear and anxiety was pervasive, regardless of documentation status. A participant who works exclusively with refugees, asylees, and parolees shared that while their clients are supposedly exempt from the worst of the anti-immigrant policies being implemented by the Trump administration… It has not stopped them [those clients] from being afraid of deportation, …of fear of the police, and fear of speaking out in public or political spaces because the threat of retribution is always there.

This participant also reported that the fear prevented these families from “applying for public benefits for which they are eligible.” Moreover, fear of family separation due to deportation was commonly reported by the participants as a prevailing change since the start of the Trump administration.

### Impact of COVID-19

The third theme COVID-19's impacts on Latino clients, including those associated with financial security, mental health, and service access barriers. One participant reported that Latino clients have experienced “increased financial needs as many have lost jobs due to the pandemic or are unable to work due to school closing.” This participant expanded on the statement, noting the intersecting harm of exclusionary immigration policies during the pandemic, mentioning that many of their Latino clients “do not qualify for government stimulus or public benefits, and many rental assistance programs require a social security number.”

Related to mental health, participants emphasized that the impact of COVID-19 was even worse than what they had observed as Latino communities coped with the Trump administration's anti-immigrant rhetoric. Multiple participants (*n* = 23) indicated witnessing increased symptoms of anxiety, depression, and suicidality among their Latino clients. Participants largely attributed elevated mental health concerns to “social isolation,” “unemployment,” and “problems with finances” related to the pandemic.

Participants also noted that new barriers to accessing health services emerged due to COVID-19. Nearly half of participants (*n* = 21) reported that their agencies shifted to telehealth service delivery, a move that presented numerous access issues for Latino clients, especially limited knowledge of and access to the required technology. One participant summarized these issues, “it is harder for us to get families signed up for telehealth because the technology instructions and interface are often only in English. Moreover, many of my Latino clients are struggling to access resources remotely because of internet connections.”

### Strategies to Enhance Service Delivery to Latino Communities

The final theme centered on strategies to strengthen service delivery to Latino clients. Participants highlighted the importance of both organizational factors, such as safety, and provider-specific factors, including cultural humility and linguistic competence, for improving services. One participant emphasized the importance of safety specific to the context of recent exclusionary immigration policies,

Latino/a immigrants need to feel safe when working with you. That is, they feel that their information is safe, and only being used for the purpose desired and not going to be used against them. They need safe immigration and judgment-free zones.

Survey participants also highlighted the value of offering services in the community. One participant noted that they started a mobile COVID-19 testing unit to assist with accessible testing during the pandemic.

Additional strategies were identified, including the importance of cultural humility, provision of linguistically competent services, and drawing on Latino cultural, family, and community strengths in service provision. As a participant summarized, providers must deliver “beneficial services that respect the dignity of each person and provide culturally and linguistically appropriate services.” One participant summarized that service provision strategies should be designed toward the overarching goal of “ensuring that Latino communities are able to experience the world with as few barriers as possible, whether those be linguistic, cultural, or socioeconomic.”

## Discussion

Findings provide valuable information related to provider-perceived experiences of Latino immigrants in the recent contexts of exclusionary immigration policies and COVID-19. More specifically, findings expand upon the current body of literature by highlighting Latino community strengths, as well as the impacts of key factors in two Southeastern, new-destination, exclusionary policy states that have imposed substantial stress on Latino communities. In regard to strengths, results were reflective of the literature by highlighting multiple strengths among Latinos. Notable was the subtheme of resilience. Resilience, which exists at individual, family, and community levels (63, 64), reflects the ability to fare well when facing stressors by buffering the harmful effects of stress on health outcomes (65–68). Research with Latino individuals suggests that resilience often stems from a sense of purpose and ethnic pride in helping their family and community members (69). For example, the core characteristics of familism, such as warmth, cohesion, and commitment to and support for one another, are evidenced by components of family-level resilience (70, 71).

Latino strength and resilience are also reflected in ethnic pride, which is a positive cognitive-emotional affiliation with one's ethnic and/or cultural community (72). Ethnic pride is associated with lower risk of depression and substance use (72–74). Latino discourse often reflects ethnic pride through culturally-specific proverbs, or dichos, which are typically used to communicate encouragement and support and to propel self-reflection (75).

Some additional literature-supported sources of strength and resilience that did not emerge in our findings include personalism, respect, and collectivism. Personalism entails engagement in relationships that are caring, trusting, supportive, and authentic (76, 77). The trust set forth by personalism establishes safe interactions in which individuals highly prioritize each other and relationships over other factors, such as personal gain or winning a disagreement (78).

Respect refers to a cultural value that guides interaction based on the positionality of those with whom one is interacting (79, 80). This value helps to keep positive interactions and reduce discord in families and in one's community (81). Collectivism encourages Latinos to perceive their own identities and responsibilities in the context of the group (82). Collectivism can serve as a protective factor for wellbeing, predicting, for example, improved outcomes in substance use disorder treatment (83). Collectivism in combination with familism have been found to predict improved physical and mental health outcomes among Latinos (84–87). Findings of this study and the existing body of literature identify multiple sources of resilience and recognize the value of resilience among Latinos.

In regard to the Trump administration, findings pointed to fear and anxiety as two impacts on Latino communities, which reflects existing literature on the detrimental effects of exclusionary immigration policies. Across the U.S., Latino communities' stress and psychological trauma was found to escalate under the Trump administration (88–92). During his campaign, former President Trump negatively targeted not only the US-Mexico border but also the Mexican and Central American individuals who cross that border. Targeted comments referenced building a wall at the US-Mexico border and inaccurately referred to Mexican immigrants as criminals and rapists coming from the most dangerous nation (5, 6). Evidence suggests that Trump's comments prompted increased anti-Latino discrimination and hate crimes within the U.S. (93). This evidence was reflected in findings of this study, as 54% of participants reported that their Latino clients experienced an increase in “flagrant discrimination and verbal abuse” since the start of the 2016 Presidential campaign.

Many Latinos are members of mixed-status families, with some members being U.S. citizens or lawful permanent residents and others unauthorized immigrants. It is estimated that 8.2 million households in the U.S. have at least one unauthorized member (94), and this number is expected to increase. Ultimately, the estimated 12 million “undocumented” Latinos and their family members (95) experience a persistent fear of deportation, along with the related fears of family separation and return to an unsafe home nation (7, 9, 87). Under the Trump administration, proposed and enacted legislation promoted increased border detentions, family separations, and enduring immigration-related insecurities (6, 96–98). For example, ending the Temporary Protected Status of Central Americans, a freeze on new applications for DACA, and initiating the Migrant Protection protocols all contributed to the overall sentiment of oppression and discrimination against Latino immigrant subgroups (4, 6, 99). For Latino children and youth growing up in the U.S., they not only had to live with the chronic stress and fear of parents being taken away, but they also experienced disruptions in their family functioning and routines as a result of their families' fear (100).

Consistent with existing research, our study found that Latinos experienced increased fear and mental health symptoms associated with hostile immigration policies (8, 101). Findings also suggest that this escalation in fear has led to a decrease in formal help-seeking behaviors among Latino communities. This finding is alarming in that Latinos experiencing heightened mental health disorders might be avoiding services altogether or withholding details of their symptoms from providers.

In terms of COVID-19, study findings raised concerns related to financial implications, mental health, and physical health. Participants' statements underscore the representation in practice settings of pandemic-related stressors that have been noted in the literature. Relative to other racial/ethnic groups, this pandemic disproportionately harmed Latino communities through elevated risk of contracting COVID-19 and its associated adverse financial and mental health implications (12, 102–104). In regard to adverse financial implications, a higher proportion of Latinos (8.6%) lost their jobs due to the pandemic than did non-Latino Whites [5.7%; (104)]. One reason for this disparity is that Latinos were overrepresented in employment types, such as in hospitality, tourism (e.g., hotels), and groundskeeping, that closed early in the pandemic (104–106).

Compared to non-Latino Whites, Latinos were 1.6 times more likely to be diagnosed with COVID-19 during the height of the pandemic, 2.5 times more likely to be hospitalized, and 2.1 times more likely to die (107). Among Latino adults with a severe illness, 64.5% live in a household with a member who had to work outside of the home during the pandemic, compared to non-Latino Black (56.5%) and White (46.6%) adults. An estimated 74% of Latino adults who are immigrants work in essential jobs such as food security and critical infrastructure, which is a greater proportion than in the general immigrant (69%) or native-born adult (65%) populations. These requirements mean that Latino adults were more likely to be exposed to COVID-19 and could more readily take it to their families.

As results indicate, the pandemic also exacerbated mental health concerns, which were already elevated as a result of Trump-era exclusionary immigration policies (19, 102, 108). With heightened financial and health risks, an increase in mental health symptoms was not surprising, especially since personal worth and success may disproportionately affect Latino mental health. For example, Chu et al. (109) found a relationship between financial hardship and suicide risk to be significantly stronger among Latino adults, and especially males, than non-Latino Whites. Another contributing factor was potentially higher family conflict and reduced family cohesion during a period when most of society was being asked to isolate and was not easily able to travel domestically or internationally to visit loved ones (110, 111). Also, religious engagement, which is a known source of Latino resilience (109), may have been reduced as faith centers moved to remote forms of engagement.

Findings specifically suggest that Latino clients experienced higher rates of anxiety, depression, and suicidality. These three mental health problems have been increasing among Latinos in the U.S. for more than a decade (44, 56) and have likely escalated with COVID-19 (107). Both existing literature and study findings suggest that mental health needs intensified during the pandemic, but also that access to those needed services declined for Latinos living in Georgia and Tennessee.

Related to service provision, findings bring attention to key access barriers faced during the pandemic. Latinos routinely encounter access barriers that include language, transportation, and lack of insurance, among others (14, 112–114). During the pandemic, existing barriers became more pertinent, and new barriers emerged. Undocumented immigrants largely lacked access to COVID-19 testing and treatment, while even documented family members may have avoided seeking services to protect undocumented loved ones from deportation (9, 11, 115, 116). Further, the hardship associated with employment loss may have intensified the already present financial barriers that many Latinos face to accessing services (117, 118). Latinos may have also encountered technology-related barriers as formal and informal interactions across the country shifted to video conference formats (119, 120). Lack of access to necessary technology and stable internet connections were identified as barriers for Latinos and other immigrant communities (121, 122).

Findings highlighted the importance of establishing a safe environment to engage Latino clients. Along this line, existing literature suggests the importance of organizations establishing safe zones in which immigrants are not at risk of immigration enforcement (123, 124). Also reflective of existing literature, study findings also point to the role of cultural humility and linguistic competence when serving Latino clients (111, 125). Another key strategy included drawing on individual, family, and community based strengths, such as familism and the value placed on community (126–128).

### Limitations

Several limitations from this study must be noted. The responses in this study were embedded in a survey study that included discrete open-ended questions. Because of this approach, we were not able to elicit additional comments or ask clarifying questions about the information provided. The qualitative responses were not asked in a way that allowed for differentiation between age, nativity, or origin among those who were immigrants. Such differentiation might have yielded discrete themes by demographic characteristics. The use of targeted and snowball sampling strategies means that the responses might not be representative of other providers in Tennessee and Georgia. Despite the limitations, findings yield meaningful implications for understanding and serving Latino communities in new destination, exclusionary policy states both in the Southeast and beyond.

### Implications

Findings can inform micro- and macro-level strategies for effectively serving Latino communities. At a micro level, providers can benefit from understanding key Latino community values of familism, personalism, respect, and collectivism. Some scholars suggest that personalism must be recognized and valued in service settings, in which provider-client relationships can influence client trust, willingness to share sensitive information, and adherence to provider advice (126–128). Recognizing that Latino clients are likely to view providers as authority figures is essential to engaging in culturally-informed practices. Building a trusting relationship in which Latinos are invited to provide input and even disagree with a provider's assessment of their situation can be vital to fully assessing and conceptualizing a presenting problem and identifying feasible treatment strategies. In the current sociopolitical context, it may be tempting to view Latinos through a lens of stress and trauma, but this is a biased approach that fails to recognize the strengths and resilience of their communities. Strengthening and supporting resilience promotes a key resource among Latinos to aid in mitigating the impacts of this stress and trauma (67, 68). Through this lens, providers can leverage the multitude of strengths among Latinos to foster improved health outcomes in Latino communities.

In addition to recognizing strengths, providers must be aware of the impacts of exclusionary immigration policies and COVID-19 on their clients. Providers can further their understanding of these factors through asking direct questions to assess effects of immigration policies and COVID-19 as key predictors for mental health and wellbeing. Due to high concerns of mental health symptoms, especially in the contexts of exclusionary policies and the pandemic, ongoing assessments of mental health status are essential. When treatment needs are identified, service provision must be designed in a way to reduce the prevalent access barriers such as cultural and linguistic barriers, fear associated with immigration enforcement, and logistical challenges such as office hours and transportation.

Multiple micro-level strategies may strengthen access and service delivery. For example, growing one's cultural competence and humility are essential to effectively engaging with and serving Latino communities (125, 129–131). Cultural competence means that providers respect clients' culture and diversity, and understand the role that cultural factors (e.g., language and beliefs) play in health outcomes (132). Cultural competence must be accompanied by cultural humility, which requires “a lifelong process of self-reflection and self-critique whereby the individual not only learns about another's culture, but one starts with an examination of her/his own beliefs and cultural identities,” (133), p. 253. Practicing cultural humility requires a continual process of learning, self-evaluation, and maintaining a respect and humility regarding one's understanding of another's culture.

Providers can also increase their familiarity with dichos as a means of support that aligns with ethnic pride (75). Use of dichos such as querer es poder, donde hay gana (roughly translated to “where there's a will there's a way”) in an appropriate and culturally sensitive manner to convey a sense of hope and encouragement (75).

Fear associated with accessing services is often multifold. Latinos who are unauthorized will be fearful of driving themselves or their family members (e.g., children) to appointments (134, 135). Other Latinos may be fearful of accessing services if they have unauthorized family members, as they fear questions that will place their loved ones at risk. To reduce fear associated with driving to access services among unauthorized immigrants, providers might consider making home visits. For clients with access to affordable technology, telehealth services may also be a valuable option. Providers can reduce other fears by avoiding questions about authorization status and by establishing safety within their setting. For example, providers can clearly state that their office is a safe setting for all immigrants, and that immigration enforcement is never notified of any client visits.

Macro-level strategies are also fundamental to reducing access barriers. At a macro level, organizations can establish safe zones, which provide protection against immigration enforcement toward undocumented immigrants seeking needed services (123, 124). In addition to advocating for safe zones within their local communities (136, 137), organizations can set forth policies that ensure that undocumented immigrants are safe from being reported or from encountering immigration enforcement activity in their agencies. This strategy will not only aid in safeguarding undocumented immigrants but might also increase service utilization among documented and U.S.-born Latinos who know that their community members are being protected by an agency (116, 138).

Provision of services within local communities, as well as offering extended hours, are also organizational approaches found to improve access to care (139–141). In addition to helping services to be available from a logistical standpoint, organizations can foster an environment in which providers have opportunities to learn about cultural responsiveness, sources of resilience, and best practices with Latino communities (111, 125, 129, 140). Within this context, organizations might provide formal trainings, either in person or online, to increase knowledge, cultural competence, and cultural humility related to serving Latino communities. Multiple trainings are accessible online through organizations such as National Latino Behavioral Health Association (NLBHA), National Hispanic and Latino Prevention Technology Transfer Center (142, 143). Providers who uphold a strong knowledge-base and understanding of cultural norms, especially areas of strength and resilience, may have a greater ability to build trust and serve Latinos through a strengths-based lens (125).

Linguistically competent service provision is a key component of culturally competent care and requires having services available in a client's preferred language. While use of interpreters and language lines can serve an essential role in the absence of fluent or native language speakers, they are substantially inferior to having language concordance in service provision (144). Scholars have found that language discordance reduces client understanding of their diagnoses and treatment plans and results in lower client satisfaction (145–148). Organizations might consider incentives for language proficiency when hiring new providers and offer language training to providers who serve clients with limited English proficiency.

Access barriers among Latino community members are profound and must be addressed through a multi-pronged approach. While these implications can be applied across settings, community-specific strategies are also essential to addressing the idiosyncratic needs within a given community.

## Conclusion

Latinos, and especially those in new destination and exclusionary policy states, such as Tennessee and Georgia, have experienced novel stressors and sources of trauma since the 2016 Presidential campaign, and throughout the COVID-19 pandemic. Concurrently, they encountered heightened service access barriers, including a lack of sufficiently trained providers to deliver culturally-informed services. Through exploration of the literature and survey responses submitted by local providers, we presented data on four themes that are pertinent to culturally-informed engagement with Latino clients: Latino communities' strengths, impact of the Trump administration, impact of COVID-19, and strategies to enhance service delivery to Latino communities. Study findings highlight individual, family, and community sources of resilience, in addition to the stressors among Latinos associated with Trump-era immigration activity and COVID-19. Key micro and macro level strategies that include establishing safe spaces, trust, and culturally and linguistically competent services are highlighted as vital to effectively working with Latino communities. Future research should expand to other new destination states and explore perceptions of both providers and Latino community members.

## Data Availability Statement

The raw data supporting the conclusions of this article will be made available by the authors, without undue reservation.

## Ethics Statement

The studies involving human participants were reviewed and approved by University of Tennessee, Knoxville; University of Georgia. Written informed consent for participation was not required for this study in accordance with the national legislation and the institutional requirements.

## Author Contributions

MH served as the principal investigator for the study and led the writing of the publication. TV-O, PJ-M, and JM were each co-PIs on the original study. TV-O helped with data analysis. All authors contributed through manuscript writing, review, and edits. All authors contributed to the article and approved the submitted version.

## Conflict of Interest

PJ-M was employed by TSNE Missionworks. The remaining authors declare that the research was conducted in the absence of any commercial or financial relationships that could be construed as a potential conflict of interest.

## Publisher's Note

All claims expressed in this article are solely those of the authors and do not necessarily represent those of their affiliated organizations, or those of the publisher, the editors and the reviewers. Any product that may be evaluated in this article, or claim that may be made by its manufacturer, is not guaranteed or endorsed by the publisher.

## Footnotes

^1^The search for an all-encompassing term to describe individuals living in the United States from (or who trace their ancestry to) Cuba, Mexico, Puerto Rico, and South and Central America continues. For this article, we follow the National Institutes of Health's ethnic categorization and definition of such individuals and use the terms “Latino/a” when referring to individuals of this broad pan-ethnic group.
